# Supplementary material for: Vasomotor Dysfunction in Patients with Ischemia and Non-Obstructive Coronary Artery Disease: Current Diagnostic and Therapeutic Strategies
Source: Biomedicines. 2021 Nov 26;9(12):1774. doi: 10.3390/biomedicines9121774 (PMC8698648; doi:10.3390/biomedicines9121774)
Supplement: Supplementary file 1 [file biomedicines-09-01774-s001.zip › biomedicines-1442566-supplementary.pdf]

Supplementary Table S1. Studies on Statins in microvascular angina.

Drug (dose)	Inclusion	Design (N)	Proposed MOA /rationale	Results	Ref
**Pravastatin (40 mg/d) for 3 months**	Cardiac syndrome-X	Randomized single-blind placebo-controlled (40)	↑Endothelial NO<br>↓Endothelin levels<br>↓inflammatory & proliferative changes<br>Cellular anti-oxidative properties	↑Brachial artery FMD<br>↑exercise duration<br>↑time to ST depression (ischemic symptoms & ECG changes disappeared completely in 5 patients)	(1)
**Simvastatin (20 mg/d)**	Cardiac syndrome X*+mildly elevated total serum cholesterol	Randomized placebo-controlled (20 in each arm)	Improving systemic endothelial function	↑Brachial artery FMD<br>↑time to ST depression in treadmill test	(2)
**Fluvastatin (40 mg/d) vs Diltiazem (90 mg/d) vs their combination**	Cardiac syndrome X	Randomized non-controlled (total of 68: 23, 22, & 23 respectively)	Both drugs previously reported to improve endothelial function but combination was not evaluated	↑ TTE CFR (all groups esp. combination)<br>↓occurrence of ST depression during exercise stress test<br>↑ time to ST depression (all groups esp. combination)<br>↑NO & ↓ ET-1 (all groups esp. combination)†	(3)

* Definition includes the presence of transient perfusion defect during myocardial perfusion scintigraphy.
† NO did not increase significantly in the Diltiazem only group but NO/ET-1 did.
NO= nitric oxide; FMD= flow-mediated dilation; TTE=trans-thoracic echocardiography; CFR=coronary flow reserve; ET-1= Endothelin-1.

Supplementary Table S2. Studies on ACE-inhibitors and angiotensin receptor blockers in microvascular angina.

Drug (dose)	Inclusion	Design (N)	Proposed MOA /rationale	Results	Ref
**Enalapril (10 mg/d 2 weeks)**	Angina+ episodes of ST segment depression on 24 ECG	Randomized, single-blind, crossover, placebo-controlled (10)	Reduced rate-pressure product<br>Direct modulation of coronary	↓ST depression & angina on exercise testing	(4)

	monitoring+ most had reversible regional perfusion abnormalities at exercise on SPECT or PET+ normal CAG+ reduced CFR by PET		microvascular artery tone<br>Attenuation of sympathetic coronary vasoconstriction & of sympathetic effects on cardiac contractility	↑total exercise duration & time to ST depression<br>↓magnitude of ST depression	
**Cilazapril (2.5 bd mg 3 weeks)**	Angina pectoris+ positive ECG+ normal CAG	Randomized double-blind crossover placebo-controlled (18)	Modulation of coronary microcirculation tone	↑ total exercise time, & time to ST depression<br>↓magnitude of ST depression	(5)
**Ramipril (2.5 mg od 4 weeks)**	Angina+ positive treadmill+ negative IV ergonovine test+ normal CAG	Single arm prospective (18)	Microvascular vasodilation	↓frequency of angina/week & need for SLN<br>↑time to angina, total exercise time, maximum MET<br>↓time to ST recovery	(6)
**Ramipril (10 mg/d)+Atorvastatin (40 mg/d 6 months)***	Angina+ exercise ischemic ST depression+ exercise SPECT reversible perfusion abnormalities+ normal CAG+ negative IV Ergonovine test	Randomized prospective single-blind placebo-controlled (45)	Antioxidant & anti-inflammatory properties→restore endothelial function<br>Additional: ↑CFR (bradykinin-mediated, NO-dependent)	↑QoL (exercise duration & SAQ)<br>↓Superoxide dismutase levels<br>↑Brachial artery flow-dependent endothelium-mediated dilation<br>↑exercise stress test duration & ↓angina & ST depression	(7)
**Ramipril (up to 10 mg)**	Angina+ no epicardial stenosis >50%+ CFVR<2.2†	1:1 randomized double-blind placebo-controlled (63)	To explore whether Ramipril has a direct effect on the microvasculature beyond BP lowering	No significant effect on CFVR or symptoms compared to placebo	(8)
**Enalapril (5 mg bd 8 weeks)**	Angina+ positive treadmill test+ normal CAG+ negative ergonovine	Randomized double-blind placebo-controlled (20)	ACEi reduces ADMA→↑NO bioavailability	↓angina & SLN use<br>↑ invasive Doppler CFR<br>↑exercise duration<br>↓plasma vWB factor & ADMA<br>↑NO level	(9)

**Quinapril (40 mg/d 1 week then 80 mg/d 15 weeks)**	No obstructive CAD ≥50% +CFR<3.0	Randomized double-blind placebo-controlled (78)	Improvement of coronary microvascular function	↓angina frequency (SAQ)‡ (2ry outcome) ↑invasive Doppler CFR if baseline ≤2.5 (1ry outcome)	(10)
**Temocapril (2 mg/d) vs Candesartan (8 mg/d)**	Type 2 diabetics (on diet or oral hypoglycemic treatment)+no overt CV disease+ CVFR ≥2.0§	Randomized single-blind	Increased availability of bradykinin rather than Angiotensin II type 1 receptor antagonism	↑CFVR (TTE) with Temocapril but not Candesartan	(11)
**Irbesartan (150 mg/d)**	Cardiac syndrome X	Randomized double-blind placebo-controlled cross-over (28)	Blocking Angiotensin II which is a powerful vasoconstrictor	Non-significant improvement in total number of ST depression on Holter monitoring and total ischemic burden on exercise	(12)

* All patients on diltiazem 180 mg/d which was kept during the study.
† Assessed by adenosine stress-echocardiography.
‡ Attributed to ACE-inhibitor treatment effect and a microvascular effect.
§ Does not follow the current standardized diagnostic criteria for microvascular angina.
SPECT=single photon emission computed tomography; PET=positron emission tomography; CAG= coronary angiography; QoL= quality of life; SAQ= Seattle Angina Questionnaire; CFVR= coronary flow velocity reserve; BP=blood pressure; ADMA= asymmetric dimethylarginine; SLN= sublingual nitroglycerin; vWB= von Willebrand; CV= cardiovascular.

Supplementary Table S3. Studies on Beta-blockers in microvascular angina.

Drug (dose)	Inclusion	Design (N)	Proposed MOA /rationale	Results	Ref
**Acebutolol (400 mg/d) vs Verapamil (80 mg qid) for 4 weeks/each treatment**	Exertional angina+ positive exercise test+ normal CAG+ negative ergonovine	Randomized double-blind crossover (30)	Acebutolol: reduce inappropriate sympathetic drive Verapamil: increasing coronary microcirculatory vasodilatory capacity	Exercise testing (pressure-rate product & total exercise duration): Verapamil effective in all patients Acebutolol effective only in higher sympathetic response	(13)

**Atenolol (100 mg/d) vs Trimetazidine (20 mg tid) for 2 weeks**	"Syndrome X"	Randomized double-blind cross-over placebo-controlled (16)	Both were shown to improve ischemia to a similar extent in overt CAD	Atenolol (& not Trimetazidine) improved symptoms, exercise performance, & TTE Doppler-indices of diastolic function	(14)
**Atenolol (100 mg/d) vs Amlodipine (10 mg/d) vs Isosorbide-5-mononitrate (50 mg/d; retard) for 4 weeks each**	Cardiac syndrome X	Randomized double-blind cross-over (10)	Standard anti-ischemic actions of these drugs (e.g. antiadrenergic effects of β-blockade)	Only Atenolol ↓ frequency of angina. Both Atenolol & Amlodipine were subjectively reported to improve QoL	(15)
**Nebivolol (5 mg/d) vs Metoprolol (50 mg/d) for 12 weeks**	Cardiac syndrome X	Randomized single-blind controlled (38)	Effects on the L-arginine/NO pathway	Nebivolol ↑plasma NO, L-arginine, L-arginine/ADMA, & ↓plasma ADMA, ↑exercise duration & CCS angina class	(16)
**Nebivolol (5mg/d) for 4 weeks**	Cardiac syndrome X	Randomized controlled (20)	Nebivolol improves endothelial function & ↑NO release	↑brachial artery lumen diameter at baseline & after reactive hyperemia, but no change in FMD	(17)
**Nebivolol (5 mg/d) vs Metoprolol (50 mg/d) for 12 weeks**	Cardiac syndrome X (spasm excluded by hyperventilation)	Randomized single-blinded metoprolol-controlled (30)	Nebivolol is suggested to exert NO-releasing effects probably by ↓ROS (unlike Metoprolol)	Nebivolol (but not metoprolol) ↑exercise duration, ↓exercise-induced ischemia, ↓anginal attacks, ↓MPO activity & MDA level, ↑serum SOD activity & NO level	(18)

CAG= coronary angiography; CAD= coronary artery disease; TTE= trans-thoracic echocardiography; QoL= quality of life; NO= nitric oxide; FMD= flow-mediated dilatation; ADMA= asymmetric dimethylarginine; CCS= Canadian Cardiovascular Society; ROS= reactive oxygen species; MPO= myeloperoxidase; MDA= malondialdehyde; SOD= superoxide dismutase.

Supplementary Table S4. Studies on Calcium channel blockers in vasospastic angina.

Drug (dose)	Inclusion	Design (N)	Proposed MOA /rationale	Results	Ref
**Verapamil (40-160 mg qid) or Nifedipine (10 to 30 mg qid)**	Normal CAG +abnormal coronary vasodilator reserve*	Randomized double-blind placebo-controlled crossover, with initial lead-in open-label phase (26)	CCB are known to be potent arterial vasodilators of both coronary & systemic circulation	↓frequency & severity of angina (occurred often at night, rest, or low activity in placebo phase)<br>↓nitroglycerin consumption<br>↑exercise duration (slightly)<br>↓exercise termination with chest pain	(19)
**Nifedipine (40 to 160 mg/d)**	ECG or angiographic evidence of coronary artery spasm (with failed other therapy including nitrates & BB)	Non-randomized non-blinded (127)	Inhibiting slow calcium current responsible for vascular smooth muscle contraction	↓weekly rate of angina (complete control in 63%)<br>↓number of SLN tabs required	(20)
**Nifedipine 30-60 mg/d, Diltiazem 90-240 mg/d, Verapamil 12-320 mg/d†**	Reversible ST elevations >0.1 mV on ECGs recorded during spontaneous anginal attacks at rest	Non-randomized non-blinded survey of data from 11 participant cardiology institutes in Japan (286)‡	Antispasmodic action rather than ↓myocardial oxygen consumption	Efficacy rates were 94%, 90.8%, and 85.7%, respectively (judged by elimination or reduction of angina attacks) regardless of the absence of >50% coronary lesions‡	(21)
**Nifedipine, ISDN**	Variant vasospastic angina pectoris	Randomized double-blind (12)		Both drugs↓ anginal episodes/day compared to pretrial period (Nifedipine was more efficacious & had less uncomfortable side effects)	(22)
**Diltiazem (60 mg tid)**	Prinzmetal's angina admitted to CCU	Randomized double-blind cross-over placebo-controlled (10)		↓number of angina episodes	(23)
**Amlodipine (10 mg/d for 4 weeks, & 5-15 mg/d long-term)**	Rest angina +1 or more of: reversible ST elevation in the absence of MI, spontaneous or	Randomized double-blind placebo-controlled short-term phase (52) followed by	Assess efficacy & safety of a 2nd generation long-acting CCB with once daily dosing, based on	↓rate of anginal episodes and intake of nitroglycerin tablets, which ↓ further at long-term	(24)

	ergonovine-induced coronary artery spasm with pain &/or ischemic ST changes, ergonovine-induced reversible perfusion defect on Th-201 scintigraphy +at least 3 episodes of rest angina during run-in period	open-label long-term extension phase (29) [15 participating centers]	the well-documented efficacy of previous 1st and 2nd generation short-acting CCBs		
**Nifedipine CR (40 mg od at night) vs Benidipine (4 mg bd) for 8 weeks**	Chest pain at rest with ischemic ST changes on Holter ECG+ coronary vasospasms in ACh or ergonovine provocation tests+ spastic angina strongly suspected by attending physicians	Randomized non-blinded (30)	Comparing therapeutic effect of Nifedipine CR which is known to be effective against vasospastic angina to Benidipine which was suggested to improve prognosis. Results attributed to night dosing of Nifedipine, its NO producing action, more potent ↓ of oxidative stress	Nifedipine and not Benidipine ↓ number of symptomatic attacks & total frequency of short-acting nitrates	(25)
**Nifedipine CR (40 mg od at bedtime) vs Diltiazem R (100 mg bd) for 12 weeks**	Angina+ induction of spasm by ACh during invasive CAG and/or no significant stenosis on CAG but recorded ischemic ST changes on ambulatory ECG with pain at rest	Randomized	Comparing efficacy of once daily nifedipine CR to twice-daily diltiazem R	Both drugs equally ↓ number of angina attacks/week (at 4,8, & 12 weeks, compared to baseline) i.e. were equally efficacious	(26)
**Benidipine, Amlodipine, Nifedipine, Diltiazem**	Positive coronary spasm provocation tests	Meta-analysis of 1,997 patients §	Examine prognostic effects	Only Benidipine had lower hazard for MACE even after correction for potentially	(27)

				confounding patient characteristics ‖	
**Conventional treatments ± Nifedipine (10-60 mg/d) for 8-10 months**	Stable angina who underwent scheduled implantation of EES in left coronary arteries	Prospective 1:1 randomized single-blinded multicenter (100) [the NOVEL study]	Vasculoprotective effects mediated at least in part by inhibition of inflammatory responses & ↓ Rho-kinase pathway	↓coronary vasoreactivity to intracoronary ACh by QCA in Nifedipine compared to control group	(28)

*Identified as having typical angina pain during rapid atrial pacing before or after administration of ergonovine 0.15 mg intravenously; with smaller increase in CBF as estimated by great cardiac vein flow using the thermodilution method, but no significant changes in epicardial coronary artery diameter after ergonovine.

†15 patients were given a combination of Nifedipine and Diltiazem (either was ineffective alone).

‡ Coronary angiography carried out in 162 patients; normal/near normal coronaries (stenosis not >50%) were found in only 70 cases. Efficacy of Nifedipine & Diltiazem was compared between the group with no distinct lesions & that with >50% stenosis in only 49 cases.

§ Patients were treated with one or combination of benidipine (n=320), amlodipine (n=308), nifedipine (n=182), or diltiazem (n=960).

‖ MACE included cardiac death, myocardial infarction, heart failure, stroke, and aortic aneurysm.

CAG= coronary angiography; CCB= calcium channel blockers; BB= β-blockers; SLN= sublingual nitrate; NO= nitric oxide; ISDN= isosorbide dinitrate; CCU= coronary care unit; MI= myocardial infarction; Th-201= Thallium-201; DES= drug-eluting stents; ACh= acetyl-choline; MACE=major adverse cardiac events; EES= everolimus-eluting stents; QCA= quantitative coronary angiography

Supplementary Table S5. Studies on Nicorandil in INOCA.

Dose	Inclusion criteria	Design (N)	Proposed MOA /rationale	Results	Ref
**3-14 days**	History of typical angina+ positive exercise electrocardiograms+ positive ^{201}Tl scintigraphy+(nearly) normal CAG+ negative provocation for epicardial coronary spasm	Prospective (11; 8 M & 3 F)	↓microcirculatory vasotone, and thus ↑ CFR	Improved scintigraphy results: improved the extent score and the severity score, and also hastened the ^{201}Tl mean washout rate	(29)
**5 mg tid for 2 weeks/each phase**	CSA + ischemic ECG during exercise + normal CAG + failed ergonovine	Randomized double-blind placebo-	Direct vasodilatory effect on coronary microvasculature	↑ time to ST depression & total exercise duration, & ↓ maximum exercise ST	(30)

	provocation of spasm + invasive Doppler-based CFR <3.0	controlled cross-over (13; 10 M & 3 F)		depression, on treadmill exercise test	
**1 mg in RCA or 2 mg in LCA (on top of isosorbide dinitrate)**	<40% epicardial coronary stenosis + TIMI-2 flow in at least one major vessel	Non-randomized	Potent microvascular dilation	↓ TIMI frame count	(31)

CAG= coronary angiography; CFR= coronary flow reserve; CSA= chronic stable angina; RCA=right coronary artery; LCA=left coronary artery; TIMI=thrombolysis in myocardial infarction.

Supplementary Table S6. Studies on Ranolazine and Ivabradine in CMD.

Drug (Dose)	Inclusion	Design (N)	Proposed MOA /rationale	Results	Ref
**Ranolazine (500 mg bd for 2 weeks, further ↑ to 1000 mg bd as tolerated)**	Angina, no obstructive CAD, ≥10% ischemic myocardium on adenosine stress CMR	Randomized double-blind placebo-controlled cross-over (20 women)	Explore impact of ranolazine on CMD patients	Better SAQ<br>Trend toward higher CMR mid-ventricular MPRI<br>Patients with CFR ≤3.0 at baseline had ↑ MPRI*	(32)
**Ranolazine (375 mg bd) or Ivabradine (5 mg bd) for 4 weeks**	Effort angina+ positive EST+ normal CAG+ CFR≤2.5 by TTE+ inadequately controlled by conventional anti-ischemics	Randomized placebo-controlled (46)	Explore any action on coronary microcirculation or systemic endothelial function beyond their 1ry action (direct inhibition of SA node by Ivabradine & improvement of ventricular diastolic function by Ranolazine)	↑ SAQ & EuroQoL (both drugs but Ranolazine > Ivabradine)<br>↑ time to ST depression & EST duration (Ranolazine)<br>No effect on TTE CFR† or peripheral FMD or NMD	(33)
**Ranolazine (up to 500 mg bd, for 8 weeks)**	Angina+ ischemia on Tc-99m MIBI+ no obstructive CAD	Randomized double-blind placebo-controlled (39 M+ 19 F)	Double action: ↓ baseline CFV & ↑ hyperemic CFV	↑ CFR by TTE	(34)

**Ranolazine (500-1000 mg/d for 2 weeks/phase)**	Ischemic symptoms+ no obstructive CAD+ preserved LVEF+ CFR<2.5 by invasive Doppler or <2.0 by stress CMR	Multicenter randomized double-blind placebo-controlled cross-over (142; 96% women)	Short-term late sodium current inhibition should be effective for CMD	No difference on SAQ, diary angina, DASI, QoL, stress MPRI (CMR), volumetric diastolic PFR and tPFR (CMR)	(35)
				Subgroup analysis of 81 patients whose CMD diagnosis was strictly based on invasive Doppler CFR<2.5: Ranolazine ↓ angina & ↑ MPRI	(36)
**Ranolazine (500 mg bd for 1 week then 1000 mg bd for 3 weeks)**	Diabetic pateints with ANOCA	1:1 randomized double-blind cross-over (35)	Determine whether ranolazine quantitatively improves exercise-stimulated myocardial blood flow & cardiac function in this patient subset	Ranolazine did not change exercise-stimulated myocardial blood flow or CFR but modestly improved diastolic function.	(37)
**Ranolazine (500 mg bd for 2 weeks then 1000 mg bd for 10 weeks)**	Angina + abnormal stress test + <50% stenosis by CAG & FFR > 0.80	Randomized double-blind placebo-controlled (26)	inhibiting the ischemic cascade induced by the late sodium current	No difference in ΔSAQ or Duke Activity Status Index, ΔCFR or ΔHMR, VO2 max, peak metabolic equivalents.at 3 months.	(38)
**Ivabradine (5 mg bd for 1 week)**	Stable CAD patients undergoing diagnostic coronary angiography	Prospective (21); Doppler-based invasive CFR measured in a non-culprit vessel at baseline, then re-measured 1 week after treatment during scheduled intervention in the culprit vessel)		↓ APV at rest<br>↑ hyperemic APV<br>↑CFR<br>(when pacing to heart rate identical to that before treatment, only APV at rest reverted to baseline values but ↑ hyperemic APV was sustained so that CFR remained higher than baseline)	(39)

* Assessed in only 13 patients who had invasive Doppler-based CFR measurements.

† By adenosine and cold pressor test

CAD= coronary artery disease; CMR= cardiac magnetic resonance; CMD= coronary microvascular dysfunction; SAQ= Seattle Angina Questionnaire; MPRI= myocardial perfusion reserve index; TTE= trans-thoracic echocardiography; CAG= coronary angiography; LVEF= left

ventricular ejection fraction; EST= exercise stress test; FMD= flow-mediated dilation; NMD= nitrate-mediated dilation; CFV= coronary flow velocity; DASI= Duke Activity Status Index; QoL= quality of life; PFR= peak filling rate, tPFR= time to peak filling rate; CFR= coronary flow reserve; FFR= fractional flow reserve; Δ= change; CFR= coronary flow reserve; HMR= hyperemic microvascular resistance; VO2 max= peak oxygen consumption; APV= average peak coronary flow velocity

Supplementary Table S7. Studies on Xanthines in CMD.

**Class/Drug**	**(Possible) mechanism of action/rationale**	**Studies**	**Disadvantages & major adverse effects**
**Xanthine derivatives**	↑ischemic threshold in CMD by 2 mechanisms:<br>Inhibit vascular smooth muscle adenosine-A2 receptors→ ↓arteriolar dilation in ischemic regions where adenosine release is ↑→ ↓redistribution of blood to these regions (coronary steal).<br>↓stimulation of cardiac nerve pain fibers by adenosine→ ↓nociception.		
**Aminophylline (6 mg/Kg body weight over 15 min)**	Prevention of myocardial flow maldistribution elicited by inappropriate adenosine release during effort	Double-blind randomized placebo-controlled study of 8 patients with cardiac syndrome X. Aminophylline ↑ effort tolerance & ↓ ECG signs of ischemia (40).	
**Aminophylline (6 mg/Kg body weight IV over 15 min)**	↓coronary steal phenomenon.	In a single-blind, placebo-controlled study of 12 patients (with typical stress-induced angina & ST depression but normal CAG & no spasm with ergonovine or Ach), aminophylline ↑ exercise time, ↓degree of ST segment depression, ↓chest pain during exercise, ↑LVEF at rest, but not at peak exercise or recovery period (41).	
**Aminophylline (oral 350 or 225 mg bd according to**	Blocking the final common pathway of cardiac pain sensation at the adenosine receptor without altering the	Double-blind cross-over study of 13 patients with syndrome X: aminophylline ↑time to angina during exercise testing, total number of angina episodes, but no difference on peak exercise ST	Nausea, palpitations (2 patients)

**BMI & smoking habit, for 3 weeks)**	initial pathological stimulus responsible for adenosine release.	depression, nor frequency or duration of ST depression during Holter monitoring (42).	
**Aminophylline (6 mg/Kg IV over 15 min)**	↓ transmural myocardial steal	Study of 14 patients with syndrome X: aminophylline ↑time to ischemia on treadmill exercise test (despite tachycardia) and ↓exercise-induced chest pain, but had no effect on total exercise duration (43).	

CMD= coronary microvascular dysfunction; CAG= coronary angiography; LVEF= left ventricular ejection fraction; BMI= body mass index.

Supplementary Table S8. Novel pharmacotherapy for INOCA/ANOCA.

**Class/Drug**	**(Possible) mechanism of action/rationale**	**Studies**	**Disadvantages & major adverse effects**
**Endothelin-A receptor antagonists**	Counteract Endothelin-1 (ET-1) which increases coronary vascular tone and contributes to coronary endothelial dysfunction (44).		Larger studies needed, high cost
**Darusentan (100 mg/d for 18 days)**	Increased absolute rest flow without an increase in pressure-rate product or perfusion during hyperemia.	Increased homogeneity of resting myocardial perfusion on PET Rb-82 scans in subjects who demonstrated a low myocardial perfusion homogeneity index compared to normal volunteers (45).	
**Atrasentan (10 mg/d for 6 months)**	Improves endothelium-dependent vasodilation.	Improved CBF % change in response to ACh in a randomized double-blind study of 47 patients (46).	
**Zibotentan**	Oral endothelin A receptor-selective antagonist.	PRIZE trial (Precision Medicine With Zibotentan in Microvascular Angina; ClinicalTrials.gov Identifier: NCT04097314; expected completion date Nov. 30th 2022) is a randomized double-blind placebo-controlled cross-over multicenter trial with 1ry outcome as exercise duration on Bruce treadmill protocol, and 2ry outcomes including patient-reported outcome measures, and a CMR sub-study to elucidate effects on MBF.	

**Rho-kinase inhibitors (Fasudil)**	↑ activity of Rho-kinase causes hypercontraction of vascular smooth muscle and plays a key pathogenetic role in coronary artery spasm (by calcium sensitization of the myosin light chain in smooth muscle cells), and is pro-inflammatory.	Intracoronary pretreatment with 300 µg/min for 15 minutes prevented ACh-induced epicardial spasm and resultant myocardial ischemia compared to saline, in a study of 20 patients with vasospastic angina (47).<br>In a study of 18 patients with ACh-induced coronary microvascular spasm, pretreatment with fasudil (4.5 mg intracoronary) prevented reproduction of spasm and ischemia with a second ACh challenge (compared to saline), and improved the lactate extraction ratio (48).	Not widely available
**Phosphodiesterase (PDE)-3 inhibitors**	Anti-platelet, anti-inflammatory, and vasodilatory effects.		
**Cilostazol (up to 200 mg/d)**	↑ NO, ↓ superoxide anion	In a study of 33 patients with VSA, on diltiazem & isosorbide mononitrate for 6 months then randomized to cilostazol, aspirin, or placebo, cilostazol ↑ invasive Doppler-based CFR (49).<br>In a prospective, multicenter, nonrandomized study of 21 patients with VSA uncontrolled with nitrates & CCBs, adding cilostazol ↓ patient-recorded angina intensity & frequency (50).<br>In a randomized, double-blind, placebo-contolled trial of 50 patients with VSA despite amlodipine therapy, cilostazol ↓ patient-recorded incidence & severity of angina (51).	Headache
**Phosphodiesterase (PDE)-5 inhibitors**	Inhibition of PDE-5 which degrades cGMP, promoting vascular smooth muscle relaxation		Larger randomized double-blind trials needed especially with longer-acting agents
**Sildenafil (100 mg once)**		In a randomized double-blind study, a subgroup of 9 patients of ANOCA/INOCA received were randomized to sildenafil, isosorbide dinitrate, or placebo. Sildenafil ↑ epicardial coronary diameter and ↓ invasive Doppler-derived CVR with	

		ACh, & ↓ ST depression at peak exercise (intermediate between placebo & isosorbide dinitrate).(52).<br>In an open-label, nonrandomized, prospective cohort of 23 women with INOCA, Sildenafil ↑ CFR in patients with baseline CFR ≤2.5 (53).	
**Tricyclic antidepressants**			
**Imipramine (50 mg/d)**	Shown to be useful in chronic pain syndromes.	In a randomized double-blind placebo-controlled cross-over trial on 18 women with ANOCA who remained symptomatic despite conventional anti-anginal therapy, imipramine treatment (5 weeks/each phase) ↓ incidence of chest pain, but failed to ↑ QoL (monitored by validated health profile questionnaire) (54).	High incidence of side effects (83%) e.g. dry mouth, dizziness, and nausea

PET= positron emission tomography; CBF= coronary blood flow; ACh= acetyl-choline; CMR= cardiac magnetic resonance; MBF= myocardial blood flow; NO= nitric oxide; VSA= vasospastic angina; CCB= calcium channel blocker; CVR= coronary vascular resistance; CFR= coronary flow reserve.

Supplementary Table S9. Non-pharmacologic therapies for refractory ANOCA.

Intervention	(Possible) mechanism of action/rationale	Studies
**Enhanced external counter-pulsation (EECP)**	Improvement of endothelial function.	In a study of 30 patients with angina refractory to usual medical therapy and non-obstructive CAD, EECP improved CCS angina class and regional ischemia on pharmacologic or exercise stress testing. At nearly 1 year of follow-up 87% had sustained improvement in angina and were without MACE (55).<br>In a study of 45 patients with ANOCA & coronary slow flow* nonrandomly assigned to medical therapy only or additional 36 1-hour sessions of EECP for 8 weeks, the EECP group had significant ↑ in resting & hyperemic diastolic peak flow velocity, CFR, and FMD (56).
**Spinal cord stimulation (SCS)**	Habituation to peripheral pain stimuli and ↓ excitability of the nociceptive system	SCS performed in 7 patients (4 men, 3 women) with refractory angina and normal coronary arteries, improved anginal symptoms, ↓ nitrate consumption, and exercise tolerance assessed subjectively by questionnaire and objectively by treadmill exercise testing (57).

		In a randomized cross-over study of 10 patients with cardiac syndrome X & refractory angina pectoris, SCS ↓ number, duration, severity of angina and nitrate consumption, as well as improved SAQ & VAS scores & tolerability to dobutamine stress testing (58).<br>In a prospective, controlled study of 19 patients (& 9 comparable controls) with refractory angina & cardiac syndrome X, SCS ↓ angina frequency, duration, & short-acting nitrate use at a median follow-up of 36 months, and improved functional status**†**, exercise tolerance, & ST segment changes (59).<br>In a study of 16 patients with effort angina, ST segment depression, but normal coronary arteries, SCS restored habituation to peripheral pain stimuli as assessed by recordings of cortical laser evoked potentials, which was speculated to help such patients better tolerate cardiac pain (60).
**Coronary sinus reducer (CSR)**	Creates a narrowing in the coronary sinus with a resultant backward pressure in the coronary venous system, which provokes dilatation of the subendocardial arterioles with reduction of vascular resistance, and subsequent redistribution of blood flow to these ischemic subendocardial layers.	THE COSIRA trial initially proved that implantation of a CSR improved anginal symptoms and QoL in patients with obstructive CAD with evidence of reversible myocardial ischemia who were not suitable candidates for revascularization (61).<br>The RESOURCE is an observational retrospective registry that included 658 patients from 20 centers with refractory angina, and confirmed the intra- and periprocedural safety of CSR implantation and its efficacy in reducing angina (39.7% improved by ≥2 CCS, and 76% by ≥1 class) (62).<br>COSIMA trial (COronary Sinus Reducer for the Treatment of Refractory Microvascular Angina) is underway (ClinicalTrials.gov Identifier: NCT04606459; expected primary completion date October 20, 2022). The 1ry objective is the proportion of eligible patients confirmed invasively to have CMD with refractory angina, reporting improvement in CCS angina class by ≥ 2 classes with implantation of CSR followed by optimal medical therapy (OMT) versus OMT alone.

* Based on Thrombolysis in Myocardial Infarction (TIMI) frame count method.

† Assessed by Seattle Angina Questionnaire and a visual analogue scale for quality of life.

CAD= coronary artery disease; CCS= Canadian Cardiovascular Society; MACE= major adverse cardiac events; CFR= coronary flow reserve; FMD= flow-mediated dilation; SAQ= Seattle Angina Questionnaire; VAS= visual analogue scale; CCS= Canadian Cardiovascular Society; FMD= flow-mediated dilatation.

References

1. Kayikcioglu M, Payzin S, Yavuzgil O, Kultursay H, Can LH, Soydan I. Benefits of statin treatment in cardiac syndrome-X1. Eur Heart J. 2003;24(22):1999-2005.
2. Fabian E, Varga A, Picano E, Vajo Z, Ronaszeki A, Csanady M. Effect of simvastatin on endothelial function in cardiac syndrome X patients. Am J Cardiol. 2004;94(5):652-5.
3. Zhang X, Li Q, Zhao J, Li X, Sun X, Yang H, et al. Effects of combination of statin and calcium channel blocker in patients with cardiac syndrome X. Coron Artery Dis. 2014;25(1):40-4.
4. Kaski JC, Rosano G, Gavrielides S, Chen L. Effects of angiotensin-converting enzyme inhibition on exercise-induced angina and ST segment depression in patients with microvascular angina. J Am Coll Cardiol. 1994;23(3):652-7.
5. Nalbantgil I, Onder R, Altintig A, Nalbantgil S, Kiliccioglu B, Boydak B, et al. Therapeutic benefits of cilazapril in patients with syndrome X. Cardiology. 1998;89(2):130-3.
6. Ozcelik F, Altun A, Ozbay G. Antianginal and anti-ischemic effects of nisoldipine and ramipril in patients with syndrome X. Clin Cardiol. 1999;22(5):361-5.
7. Pizzi C, Manfrini O, Fontana F, Bugiardini R. Angiotensin-converting enzyme inhibitors and 3-hydroxy-3-methylglutaryl coenzyme A reductase in cardiac Syndrome X: role of superoxide dismutase activity. Circulation. 2004;109(1):53-8.
8. Michelsen MM, Rask AB, Suhrs E, Raft KF, Host N, Prescott E. Effect of ACE-inhibition on coronary microvascular function and symptoms in normotensive women with microvascular angina: A randomized placebo-controlled trial. PLoS One. 2018;13(6):e0196962.
9. Chen JW, Hsu NW, Wu TC, Lin SJ, Chang MS. Long-term angiotensin-converting enzyme inhibition reduces plasma asymmetric dimethylarginine and improves endothelial nitric oxide bioavailability and coronary microvascular function in patients with syndrome X. Am J Cardiol. 2002;90(9):974-82.
10. Pauly DF, Johnson BD, Anderson RD, Handberg EM, Smith KM, Cooper-DeHoff RM, et al. In women with symptoms of cardiac ischemia, nonobstructive coronary arteries, and microvascular dysfunction, angiotensin-converting enzyme inhibition is associated with improved microvascular function: A double-blind randomized study from the National Heart, Lung and Blood Institute Women's Ischemia Syndrome Evaluation (WISE). Am Heart J. 2011;162(4):678-84.
11. Kawata T, Daimon M, Hasegawa R, Teramoto K, Toyoda T, Sekine T, et al. Effect on coronary flow velocity reserve in patients with type 2 diabetes mellitus: comparison between angiotensin-converting enzyme inhibitor and angiotensin II type 1 receptor antagonist. Am Heart J. 2006;151(4):798 e9-15.
12. Russell SJ, Di Stefano EM, Naffati MT, Brown O, Saltissi S. The effects of the angiotensin II receptor (type I) antagonist irbesartan in patients with cardiac syndrome X. Heart. 2007;93(2):253-4.
13. Romeo F, Gaspardone A, Ciavolella M, Gioffre P, Reale A. Verapamil versus acebutolol for syndrome X. Am J Cardiol. 1988;62(4):312-3.
14. Leonardo F, Fragasso G, Rossetti E, Dabrowski P, Pagnotta P, Rosano GM, et al. Comparison of trimetazidine with atenolol in patients with syndrome X: effects on diastolic function and exercise tolerance. Cardiologia. 1999;44(12):1065-9.
15. Lanza GA, Colonna G, Pasceri V, Maseri A. Atenolol versus amlodipine versus isosorbide-5-mononitrate on anginal symptoms in syndrome X. Am J Cardiol. 1999;84(7):854-6, A8.
16. Sen N, Tavil Y, Erdamar H, Yazici HU, Cakir E, Akgul EO, et al. Nebivolol therapy improves endothelial function and increases exercise tolerance in patients with cardiac syndrome X. Anadolu Kardiyol Derg. 2009;9(5):371-9.
17. Kayaalti F, Kalay N, Basar E, Mavili E, Duran M, Ozdogru I, et al. Effects of nebivolol therapy on endothelial functions in cardiac syndrome X. Heart Vessels. 2010;25(2):92-6.

18. Erdamar H, Sen N, Tavil Y, Yazici HU, Turfan M, Poyraz F, et al. The effect of nebivolol treatment on oxidative stress and antioxidant status in patients with cardiac syndrome-X. Coron Artery Dis. 2009;20(3):238-4.
19. Cannon RO, 3rd, Watson RM, Rosing DR, Epstein SE. Efficacy of calcium channel blocker therapy for angina pectoris resulting from small-vessel coronary artery disease and abnormal vasodilator reserve. Am J Cardiol. 1985;56(4):242-6.
20. Antman E, Muller J, Goldberg S, MacAlpin R, Rubenfire M, Tabatznik B, et al. Nifedipine therapy for coronary-artery spasm. Experience in 127 patients. N Engl J Med. 1980;302(23):1269-73.
21. Kimura E, Kishida H. Treatment of variant angina with drugs: a survey of 11 cardiology institutes in Japan. Circulation. 1981;63(4):844-8.
22. Ginsburg R, Lamb IH, Schroeder JS, Hu M, Harrison DC. Randomized double-blind comparison of nifedipine and isosorbide dinitrate therapy in variant angina pectoris due to coronary artery spasm. Am Heart J. 1982;103(1):44-9.
23. Pesola A, Lauro A, Gallo R, Madeo A, Cosentino G. Efficacy of diltiazem in variant angina. Results of a double-blind crossover study in CCU by Holter monitoring. The possible occurrence of a withdrawal syndrome. G Ital Cardiol. 1987;17(4):329-39.
24. Chahine RA, Feldman RL, Giles TD, Nicod P, Raizner AE, Weiss RJ, et al. Randomized placebo-controlled trial of amlodipine in vasospastic angina. Amlodipine Study 160 Group. J Am Coll Cardiol. 1993;21(6):1365-70.
25. Oikawa Y, Matsuno S, Yajima J, Nakamura M, Ono T, Ishiwata S, et al. Effects of treatment with once-daily nifedipine CR and twice-daily benidipine on prevention of symptomatic attacks in patients with coronary spastic angina pectoris-Adalat Trial vs Coniel in Tokyo against Coronary Spastic Angina (ATTACK CSA). J Cardiol. 2010;55(2):238-47.
26. Higuma T, Oikawa K, Kato T, Mori Y, Kudo T, Yamamoto T, et al. Comparison of the effects of long-acting nifedipine CR and diltiazem R in patients with vasospastic angina: Aomori coronary spastic angina study. J Cardiol. 2010;56(3):354-60.
27. Nishigaki K, Inoue Y, Yamanouchi Y, Fukumoto Y, Yasuda S, Sueda S, et al. Prognostic effects of calcium channel blockers in patients with vasospastic angina--a meta-analysis. Circ J. 2010;74(9):1943-50.
28. Tsuburaya R, Takahashi J, Nakamura A, Nozaki E, Sugi M, Yamamoto Y, et al. Beneficial effects of long-acting nifedipine on coronary vasomotion abnormalities after drug-eluting stent implantation: The NOVEL study. Eur Heart J. 2016;37(35):2713-21.
29. Yamabe H, Namura H, Yano T, Fujita H, Kim S, Iwahashi M, et al. Effect of nicorandil on abnormal coronary flow reserve assessed by exercise 201Tl scintigraphy in patients with angina pectoris and nearly normal coronary arteriograms. Cardiovasc Drugs Ther. 1995;9(6):755-61.
30. Chen JW, Lee WL, Hsu NW, Lin SJ, Ting CT, Wang SP, et al. Effects of short-term treatment of nicorandil on exercise-induced myocardial ischemia and abnormal cardiac autonomic activity in microvascular angina. Am J Cardiol. 1997;80(1):32-8.
31. Sadamatsu K, Tashiro H, Yoshida K, Shikada T, Iwamoto K, Morishige K, et al. Acute effects of isosorbide dinitrate and nicorandil on the coronary slow flow phenomenon. Am J Cardiovasc Drugs. 2010;10(3):203-8.
32. Mehta PK, Goykhman P, Thomson LE, Shufelt C, Wei J, Yang Y, et al. Ranolazine improves angina in women with evidence of myocardial ischemia but no obstructive coronary artery disease. JACC Cardiovasc Imaging. 2011;4(5):514-22.
33. Villano A, Di Franco A, Nerla R, Sestito A, Tarzia P, Lamendola P, et al. Effects of ivabradine and ranolazine in patients with microvascular angina pectoris. Am J Cardiol. 2013;112(1):8-13.
34. Tagliamonte E, Rigo F, Cirillo T, Astarita C, Quaranta G, Marinelli U, et al. Effects of ranolazine on noninvasive coronary flow reserve in patients with myocardial ischemia but without obstructive coronary artery disease. Echocardiography. 2015;32(3):516-21.

35. Bairey Merz CN, Handberg EM, Shufelt CL, Mehta PK, Minissian MB, Wei J, et al. A randomized, placebo-controlled trial of late Na current inhibition (ranolazine) in coronary microvascular dysfunction (CMD): impact on angina and myocardial perfusion reserve. Eur Heart J. 2016;37(19):1504-13.
36. Rambarat CA, Elgendy IY, Handberg EM, Bairey Merz CN, Wei J, Minissian MB, et al. Late sodium channel blockade improves angina and myocardial perfusion in patients with severe coronary microvascular dysfunction: Women's Ischemia Syndrome Evaluation-Coronary Vascular Dysfunction ancillary study. Int J Cardiol. 2019;276:8-13.
37. Shah NR, Cheezum MK, Veeranna V, Horgan SJ, Taqueti VR, Murthy VL, et al. Ranolazine in Symptomatic Diabetic Patients Without Obstructive Coronary Artery Disease: Impact on Microvascular and Diastolic Function. J Am Heart Assoc. 2017;6(5).
38. Koh JS, Hung OY, Eshtehardi P, Kumar A, Rabah R, Raad M, et al. Microvascular Assessment of Ranolazine in Non-Obstructive Atherosclerosis: The MARINA Randomized, Double-Blinded, Controlled Pilot Trial. Circ Cardiovasc Interv. 2020;13(12):e008204.
39. Skalidis EI, Hamilos MI, Chlouverakis G, Zacharis EA, Vardas PE. Ivabradine improves coronary flow reserve in patients with stable coronary artery disease. Atherosclerosis. 2011;215(1):160-5.
40. Emdin M, Picano E, Lattanzi F, L'Abbate A. Improved exercise capacity with acute aminophylline administration in patients with syndrome X. J Am Coll Cardiol. 1989;14(6):1450-3.
41. Yoshio H, Shimizu M, Kita Y, Ino H, Kaku B, Taki J, et al. Effects of short-term aminophylline administration on cardiac functional reserve in patients with syndrome X. J Am Coll Cardiol. 1995;25(7):1547-51.
42. Elliott PM, Krzyzowska-Dickinson K, Calvino R, Hann C, Kaski JC. Effect of oral aminophylline in patients with angina and normal coronary arteriograms (cardiac syndrome X). Heart. 1997;77(6):523-6.
43. Yesildag O, Yazici M, Yilmaz O, Ucar R, Sagkan O. The effect of aminophylline infusion on the exercise capacity in patients with syndrome X. Acta Cardiol. 1999;54(6):335-7.
44. Ford TJ, Rocchiccioli P, Good R, McEntegart M, Eteiba H, Watkins S, et al. Systemic microvascular dysfunction in microvascular and vasospastic angina. Eur Heart J. 2018;39(46):4086-97.
45. Johnson NP, Gould KL. Physiology of endothelin in producing myocardial perfusion heterogeneity: a mechanistic study using darusentan and positron emission tomography. J Nucl Cardiol. 2013;20(5):835-44.
46. Reriani M, Raichlin E, Prasad A, Mathew V, Pumper GM, Nelson RE, et al. Long-term administration of endothelin receptor antagonist improves coronary endothelial function in patients with early atherosclerosis. Circulation. 2010;122(10):958-66.
47. Masumoto A, Mohri M, Shimokawa H, Urakami L, Usui M, Takeshita A. Suppression of coronary artery spasm by the Rho-kinase inhibitor fasudil in patients with vasospastic angina. Circulation. 2002;105(13):1545-7.
48. Mohri M, Shimokawa H, Hirakawa Y, Masumoto A, Takeshita A. Rho-kinase inhibition with intracoronary fasudil prevents myocardial ischemia in patients with coronary microvascular spasm. J Am Coll Cardiol. 2003;41(1):15-9.
49. Watanabe K, Ikeda S, Komatsu J, Inaba S, Suzuki J, Sueda S, et al. Effect of cilostazol on vasomotor reactivity in patients with vasospastic angina pectoris. Am J Cardiol. 2003;92(1):21-5.
50. Yoo SY, Song SG, Lee JH, Shin ES, Kim JS, Park YH, et al. Efficacy of cilostazol on uncontrolled coronary vasospastic angina: a pilot study. Cardiovasc Ther. 2013;31(3):179-85.
51. Shin ES, Lee JH, Yoo SY, Park Y, Hong YJ, Kim MH, et al. A randomised, multicentre, double blind, placebo controlled trial to evaluate the efficacy and safety of cilostazol in patients with vasospastic angina. Heart. 2014;100(19):1531-6.
52. Halcox JP, Nour KR, Zalos G, Mincemoyer RA, Waclawiw M, Rivera CE, et al. The effect of sildenafil on human vascular function, platelet activation, and myocardial ischemia. J Am Coll Cardiol. 2002;40(7):1232-40.

53. Denardo SJ, Wen X, Handberg EM, Bairey Merz CN, Sopko GS, Cooper-Dehoff RM, et al. Effect of phosphodiesterase type 5 inhibition on microvascular coronary dysfunction in women: a Women's Ischemia Syndrome Evaluation (WISE) ancillary study. Clin Cardiol. 2011;34(8):483-7.
54. Cox ID, Hann CM, Kaski JC. Low dose imipramine improves chest pain but not quality of life in patients with angina and normal coronary angiograms. Eur Heart J. 1998;19(2):250-4.
55. Kronhaus KD, Lawson WE. Enhanced external counterpulsation is an effective treatment for Syndrome X. Int J Cardiol. 2009;135(2):256-7.
56. Luo C, Liu D, Wu G, Hu C, Zhang Y, Du Z, et al. Effect of enhanced external counterpulsation on coronary slow flow and its relation with endothelial function and inflammation: a mid-term follow-up study. Cardiology. 2012;122(4):260-8.
57. Lanza GA, Sestito A, Sandric S, Cioni B, Tamburrini G, Barollo A, et al. Spinal cord stimulation in patients with refractory anginal pain and normal coronary arteries. Ital Heart J. 2001;2(1):25-30.
58. Lanza GA, Sestito A, Sgueglia GA, Infusino F, Papacci F, Visocchi M, et al. Effect of spinal cord stimulation on spontaneous and stress-induced angina and 'ischemia-like' ST-segment depression in patients with cardiac syndrome X. Eur Heart J. 2005;26(10):983-9.
59. Sgueglia GA, Sestito A, Spinelli A, Cioni B, Infusino F, Papacci F, et al. Long-term follow-up of patients with cardiac syndrome X treated by spinal cord stimulation. Heart. 2007;93(5):591-7.
60. Sestito A, Lanza GA, Le Pera D, De Armas L, Sgueglia GA, Infusino F, et al. Spinal cord stimulation normalizes abnormal cortical pain processing in patients with cardiac syndrome X. Pain. 2008;139(1):82-9.
61. Verheye S, Jolicoeur EM, Behan MW, Pettersson T, Sainsbury P, Hill J, et al. Efficacy of a device to narrow the coronary sinus in refractory angina. N Engl J Med. 2015;372(6):519-27.
62. Ponticelli F, Khokhar AA, Leenders G, Konigstein M, Zivelonghi C, Agostoni P, et al. Safety and efficacy of coronary sinus narrowing in chronic refractory angina: Insights from the RESOURCE study. Int J Cardiol. 2021;337:29-37.
